# Effect of the surface coating of carbonyl iron particles on the dispersion stability of magnetorheological fluid

**DOI:** 10.1038/s41598-024-61934-2

**Published:** 2024-05-18

**Authors:** Fang Chen, Jie Zhang, Zhenggui Li, Shengnan Yan, Wangxu Li, Zhaoqiang Yan, Xiaobing Liu

**Affiliations:** 1 Key Laboratory of Fluid Machinery and Engineering (Xihua University), Ministry of Education, Chengdu, 610039 People’s Republic of China; 2 School of Energy and Power Engineering, Lanzhou University of Technology, Lanzhou, 730050 People’s Republic of China; 3 Zigong Zhaoqiang Sealing Products Industrial Co., Ltd., Zigong, 643000 People’s Republic of China

**Keywords:** Magnetorheological fluid, Carbonyl iron particle, Hydrochloric acid etching, Chain length, Silane coupling agent, Dispersion stability, Chemistry, Materials chemistry, Polymer chemistry, Surface chemistry, Chemical synthesis

## Abstract

The dispersion stability of carbonyl iron particle (CIP)-based magnetorheological fluid (MRF) is improved by CIP, which particle is etched with hydrochloric acid (HCl) to form porous structure with many hydroxyl groups and subsequently coated with silane coupling agents that have varying chain lengths. The microstructures, coating effect and magnetism of the CIPs were examined using the Scanning Electron Microscopy, Automatic Surface and Porosity Analyzer (BET), Fourier-Transform Infrared Spectroscopy, Thermogravimetric Analysis and Vibrating Sample Magnetometer. Furthermore, the rheological properties and dispersion stability of the MRFs were assessed using a Rotating Rheometer and Turbiscan-lab. The results revealed that the nanoporous structure appeared on the CIPs and the specific surface area increased remarkably after being etched by hydrochloric acid. Additionally, as the chain length of the silane coupling agent increases, the coated mass on the particles increases, the the density and the saturation magnetization of particles decreased, and the coated particles with different shell thicknesses were obtained; without a magnetic field, the viscosity of MRF prepared by coated particles increase slightly, due to the enhancement of special three-dimensional network structure; under a magnetic field, the viscosity of the MRF decreased distinctly; the sedimentation rate of MRF decreased from 58 to 3.5% after 100 days of sedimentation, and the migration distances of the MRFs were 22.4, 3.7, 2.4, and 0 mm, with particle sedimentation rates of 0.149, 0.019, 0.017, and 0 mm/h, respectively. The MRF with high dispersion stability was obtained, and the etching of CIP by HCl and the proper chain length of the coating of silane coupling agent were proved effective manners to improve the dispersion stability of MRF.

## Introduction

Magnetorheological fluids (MRFs) are primarily composed of magnetic particles, a carrier medium, and additives. Without a magnetic field, the magnetic particles are randomly distributed in the carrier medium, and the MRF is a free-flowing Newtonian fluid; under a magnetic field, chain structures of the magnetic particles form along the direction of the magnetic field within milliseconds, the solid-like Bingham plastic material property of the MRF appears, and the wide variation range of the yield stress is between 20 and 100 kPa^1–3^. As an intelligent controllable material, MRF is widely used in damping, transmission devices, sealing, polishing, and medical equipment, etc., owing to its unique magnetorheological effect^4–8^. The commercialization of MRF and related equipment has advanced significantly, but the sedimentation issue of MRF introduced by the density mismatch between the magnetic particles and carrier medium has severely impeded further advancements in the MRF performance and related equipment^9–11^.

Several solutions have been proposed to improve the dispersion stability of the MRF, such as the addition of micron-scale graphene oxide, submicron/nano-particles to form a double-dispersed phase^12–15^, the coating of shell structures on the particle surfaces^16^, and use of a high-viscosity fluid as a continuous phase^17^, of which the surface coating of CIP is considered to be a highly effective method to reduce the sedimentation rate of the MRF^16,18–20^. When cholesteryl chloroformate was coated on the surface of CIP, the density of the coated particles decreased, and the sedimentation rate of the MRFs were 65% prepared by pure CIP and 45% by coated particles after 30 h^21^. The CIP was coated with polystyrene foam for reducing the density of the particle, and the sedimentation rate of the MRFs were 80% prepared by pure CIP and 30% by coated particles after 24 h^19^. ZnO/CIP core–shell particles were obtained by coating zinc oxide on the CIP surface, which reduced the density of CIP, and the sedimentation rate of the MRFs were 70% prepared by pure CIP and 45% by coated particles after 30 h^22^. The graphene oxide (GO) were coated on the CIP, and the sedimentation rate of the MRFs were 22% prepared by pure CIP and 4% by coated particles after 60 days^23^. The density reduction of magnetic particles is known to be effective for improving the stability of MRFs; in addition, the improvement of the compatibility between the coated particles and carrier medium is also significantly useful^24,25^. A recent study by Ronzova et al. reported that for particles coated by (3-amino-propyl)triethoxy-silane (APTES), tetraethoxy silane (TEOS), vinyltrimethoxy-silane (VIN), and bis[3(3-methoxy-silane)propyl]amine (BIS), the dispersion stability of MRF prepared by VIN coated particles is the best, and the sedimentation rate of the MRFs were 70% prepared by pure CIP and 20% by coated particles after 100 min, owing to the fact that the compatibility of the VIN coating with silicone oil is higher than that of other coatings^26^. Consequently, the type of coated silane coupling agent has a significant impact on the MRF stability.

Furthermore, a double-layer coating of particle coating has been reported to be usefully for the dispersion stability of MRFs. Fang et al. used carbon nanotubes coated with polyaniline to coat the CIP, which reduced the particle density and increased the surface roughness of the particles, thus delaying the sedimentation rate of the particles in the carrier medium^27^. In double-layer coating of particles, certain silane coupling agents with functional groups are usually used as the first coating^28–31^; for example, the CIP surface was first coated with APTES and subsequently coated with poly(n-butyl acrylate), and the sedimentation rate of the prepared MRF was 20% after 30 h^32^.

Notably, the dispersion stability of the particles in the carrier medium is significantly influenced by the chain length of the polymer utilized in the coating^33^. Cheng et al. compared the sedimentation rate of CIP particles coated with octyl acyl ethylenediamine triacetate (C_7_H_15_COED_3_A), lauryl acyl ethylenediamine triacetate (C_11_H_23_COED_3_A), and stearyl acyl ethylenediamine triacetate(C_17_H_35_COED_3_A) in silicone oil. These three types of polymers have different chain lengths; the sedimentation rate of the CIP particles decreased from 2.46 × 10^−3^ to 1.33 × 10^−3^ h^−1^ as the chain length of the coating increased^25^. Belyavskii et al. demonstrated that the grafting density of the coated long chain molecules on CIP is significantly lower than that of the coated short chain molecules due to the steric hindrance^34^. Moreover, the coated multi-functional reagents of the particles,can provide the higher reactivity to the hydroxy groups at the surface of CIP than single-functional ragents, such as 1,2-bis(triethoxy-silyl)ethane, so as to enhance the grafting density^34^.

Furthermore, studies have reported that the etching of carbonyl iron particles with HCl makes the pore with sizes from 21 to 80 nm form, and makes the specific surface area of the particles increase from 1.24 to 16.92 m^2^/g^35^. Due to the interaction of the unfinished 3d orbital in Fe^3+^ with the lone pair electron in the 2p orbital of the oxygen atom in H_2_O, the particle surface exhibits Lewis acidity after the CIP is etched by HCl and the interaction reduces dissociation energy and weakens O–H bond in H_2_O molecules, resulting in chemisorption of –OH on etched particle surface^36,37^.

As previously indicated, there are several practical techniques to increase the coating effect of particles to enhance the dispersion stability of the MRFs, including etching particles with acid solutions, double-layer coatings, the coated multi-functional reagents, and coating polymers with suitable chain lengths. Although the sedimentation issue of MRF was improved, it is difficult to obtain the MRF with a long-term dispersion stability, and which is great important in various related applications.

In this study, CIP was etched firstly, and then coated with 1,2-bis(triethoxy-silyl)ethane (BTES), and subsequently coated with *n*-octyltriethoxy silane (TOS) or dodecyl trimethoxysilane (DTOS) having different chain lengths. To prepare the MRF, these particles were dispersed in polyalphaolefin synthetic oil, which has an outstanding high-temperature stability, shear stability, and low-temperature fluidity. The microstructures of the magnetic particles, rheological properties, and dispersion stabilities of the MRF were characterized, respectively. The etching and coating of the CIP particles with a suitable chain-length silane coupling agent was proved usefully to improve the MRF dispersion stability. The results of this study have significant implications for basic theoretical research and MRF-related applications.

## Experiment

### Materials and chemicals

Hydrochloric acid (HCl, AR) and anhydrous ethanol (C_2_H_5_OH, AR) were purchased from Chengdu Colon Chemical Co., Ltd. (Chengdu, China). Carbonyl iron powder (CIP, 3–6 μm) was purchased from Shanghai Xiangtian Nanomaterials Co., Ltd (Shanghai, China). 1,2-bis(triethoxy-silyl)ethane (C_14_H_34_O_6_Si_2_, BTES, purity ≥ 95%), *n*-octyltriethoxy silane (C_14_H_32_O_3_Si, TOS, purity > 97%), and dodecyl trimethoxysilane (C_15_H_34_O_3_Si, DTOS, purity > 95%) were purchased from Shanghai Maclin Technology Co., Ltd. (Shanghai, China). Polyalphaolefin synthetic oil-100 (density: 0.846 g/cm^3^, Kinematic viscosity: 1023 mm^2^/s) was purchased from Shanghai Nac Lubrication Technology Co. Ltd. (Shanghai, China). Ultra-pure water was used.

### Preparation method

#### The etching of particles

The CIP was etched with HCl prior to being coated. The CIPs were etched and the porous carbonyl iron particles (HCIP) were obtained after being etched, 40 g of CIP were ehched with 100 ml of ultrapure water and 0.5 mol/L of HCl. The mixture was agitated at 200 rpm for 10 min at 40 °C^35^.

#### The coating of HCIP

Silane coupling agents (Table 1) with different chain lengths were used to coat the HCIP.

**Table 1:** The silane coupling agents used in the coating.

Abbreviation	Reagent	Molecular formula
BTES	1,2-bis(triethoxy-silyl)ethane	(C_6_H_15_O_3_)_2_Si_2_C_2_H_4_
TOS	N-octyltriethoxy silane	(C_6_H_15_O_3_)SiC_8_H_17_
DTOS	Dodecyl trimethoxysilane	(C_3_H_9_O_3_)SiC_12_H_25_

The HCIP was placed in a mixture of 200 ml absolute ethanol and 15 ml ultrapure water, and approximately 5 g BTES was added to the mixture solution. Mechanical mixing was performed at 250 rpm for 2 h, subsequently, the single-layer coated particles (HCIP-BTES) were obtained. On this basis, approximately 6 g of TOS or DTOS were coated on HCIP-BTES, respectively, and the particles coated by a double-layer with varying alkyl chain lengths were obtained; the reaction conditions were the same as that ofthe single-layer coating. At last, all the particles were cleaned 6 times using ultra-pure water and then were dried at 80 °C for 24 h.

#### Preparation of MRF

**Figure 1:**
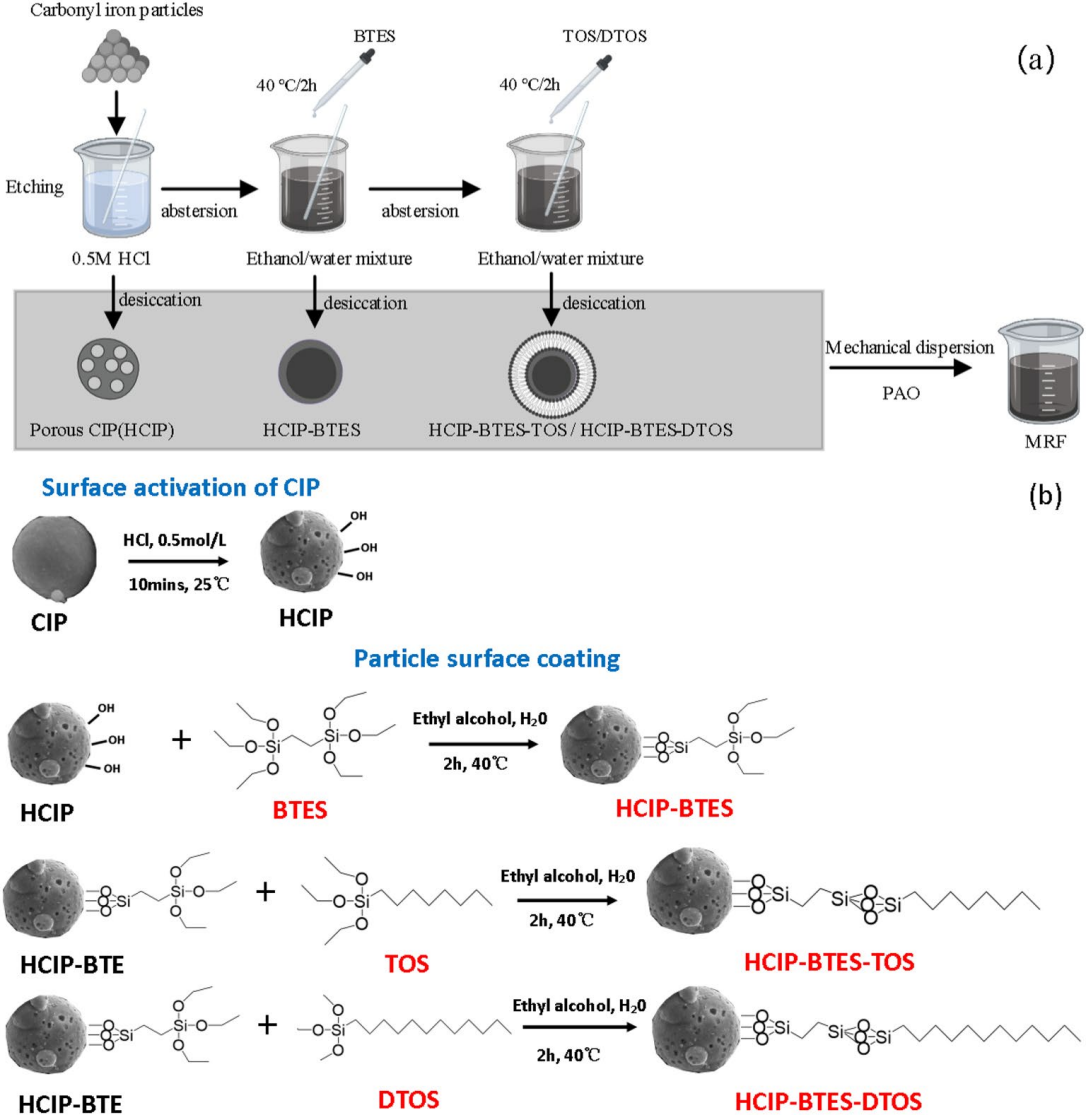
The preparation of MRF: (**a**) Preparation flow chart of MRF; (**b**) The coating mechanism diagram of particle.

Magnetic particles coated with different chain lengths were mixed in PAO, for preparing a MRF with a mass fraction of 60 wt%, mechanical stirring was conducted at 700 rpm for 2 h at room temperature. The preparation process of the particles and MRF is shown in Fig. 1.

### Characterization method

The microstructure of the CIP was characterized using Scanning Electron Microscopy (SEM, Phenom Pharos G2), and the magnifications are 20,000× and 5000× respectively. The specific surface area and porous structure characteristics of the CIP were calculated by an Automatic Surface and Porosity Analyzer (BET, Micromeritics ASAP 2460), and the removal temperature was 120 °C by nitrogen adsorption. The coating thickness of the particles was observed using Transmission Electron Microscopy (TEM, JEM-F200). A Fourier-Transform Infrared Spectroscopy (FTIR, Nicolet iS 10) was used to characterize the coating effect of silane coupling agents on the surface of the particles, with wavenumbers ranging from 400 to 4000 cm^−1^. A Thermal Gravimetric Analyzer (TGA, TA) was used to analyze the coated mass on the particle surface under the condition of a nitrogen atmosphere, the temperature range was from 30 to 600 °C, and the heating rate was 20 °C/min. A Density Meter (AccuPyc 1340) was used to test the density of the coated particles, where the amount of particles was about 100 mg, and the true volume of the particles was measured by helium as the expanding gas medium. A Vibrating Sample Magnetometer (VSM, Lake Shore 7404) was used to test the VSM curves of the magnetic particles in the applied field range of − 2.5 × 10^4^ Oe to + 2.5 × 10^4^ Oe at 25 °C. The magnetorheological properties of the MRF were measured using a Rotary Rheometer (MCR 302e Anton Paar, Austria), the measuring system is PP20/MRD/TI SN94511, the test unit is MRD170 + H-PTD220, and the magnetic field is controlled by the magnetic control system (MRD 170/1T). The sample platform is parallel plate with a shear disc gap of 1 mm, magnetic field strengths is 0, 57, and 108 kA/m, and the range of shear rate is from 0.1 to 1000 s^−1^. To analyze the dispersion stability, a static sedimentation observation method was adopted, and the MRF was placed in a 10 ml cylinder and was observed every 24 h till 100 d. The volume fraction of the supernatant in the MRF was recorded. Furthermore, the dispersion stability of the MRF was measured using Turbiscan-Lab (Formulation, France); the MRF was placed into a cylindrical glass tube, and the samples were periodically scanned from bottom to top by the beam of near-infrared light (*λ* = 880 nm), and the migration rates of the particles were derived.

## Results and discussion

### Morphology of the CIP particles

**Figure 2:**
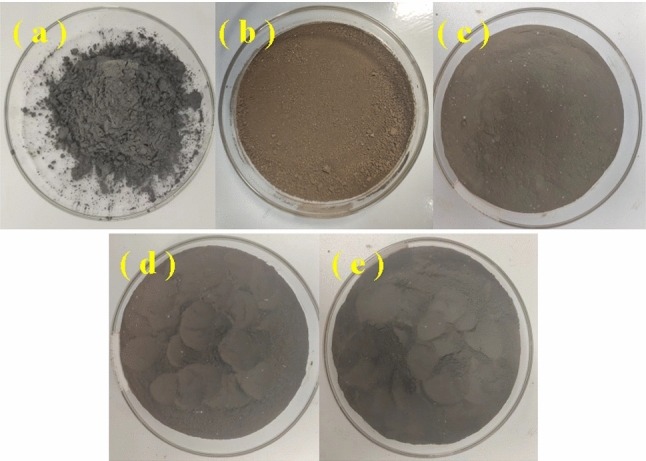
The macroscopic morphologies of CIPs: (**a**) CIP, (**b**) HCIP, (**c**) HCIP-BTES, (**d**) HCIP-BTES-TOS and (**e**) HCIP-BTES-DTOS.

The macroscopic morphologies of the particles are shown in Fig. 2. The CIP is bluish gray (Fig. 2a), whereas the HCIP is reddish-brown (Fig. 2b), caused by the oxidation reaction when particles contacted with the oxygen from air^34^. After being coated with coupling agents, the degree of reddish-brown of particles (Fig. 2c) decreased, indicating that the coating reduced the oxidation. Not only that, the color of particles gradually deepened when the chain length of the coupling agent increased (Fig. 2d and e), indicating that the silane coupling agents with various chain lengths were coated on the surface of the particles^33^.

**Figure 3:**
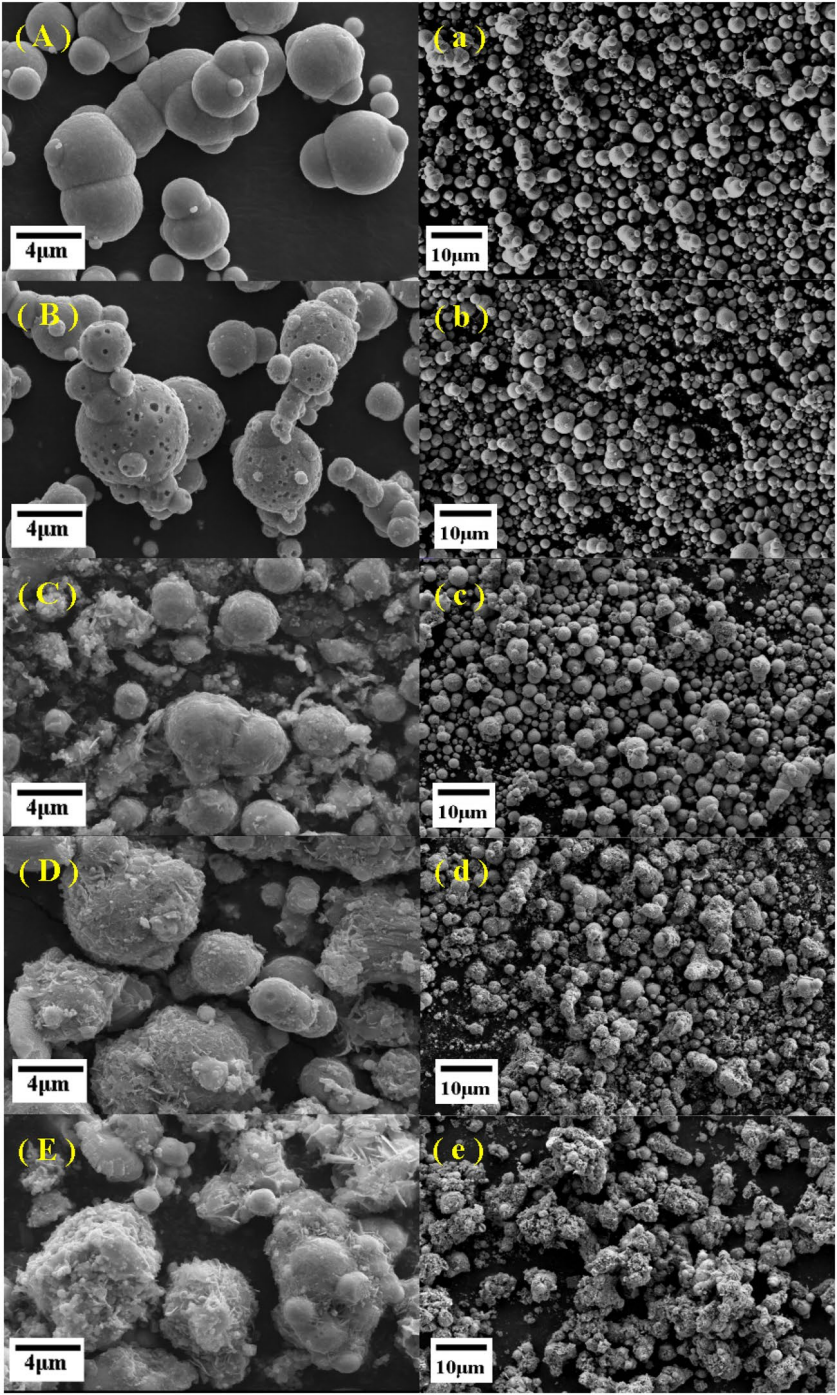
SEM images of the particles and their size distributions: (**A**) and (**a**) CIP, (**B**) and (**b**) HCIP, (**C**) and (**c**) HCIP-BTES, (**D**) and (**d**) HCIP-BTES-TOS, (**E**) and (**e**) HCIP-BTES-DTOS.

The SEM images of particles are shown in Fig. 3. The pure CIP is apparently spherical with a smooth surface (Fig. 3A) and the particles are scattered distributed^25^. Notably, the surface of the particle becomes rough, and many nanopores appeared on the surface of the HCIP (Fig. 3B), is has been reported that which will increases the specific surface area of particles and the number of –OH functional groups, and thus which will enhance the coating effect on the particle surface^35–37^. Certain coated floccules can be clearly observed on the surface of the single-layer coated particles (Fig. 3C). As the chain length of the coupling agent increased, the coated floccules shown in Fig. 3D became more coarser on the particle surface. In addition, more floccules showed in Fig. 3E, and the particles are entirely encased by the coatings, as shown in Fig. 3e^38,39^. The SEM images indicate that the silane coupling agents with different chain lengths can achieve single or double layer coating on the particles.

Table 2 shows the specific surface area of particles. In the table, the *S*_BET_ is the specific surface area of CIP, the *S*_BJH_ is the specific surface area of nanopores, and the *V*_BJH_ is the specific volume of nanopores. The *S*_BET_ of the purchased CIP is 0.474 m^2^/g, after being etched with HCl, the *S*_BET_ increases to 37.773 m^2^/g, and the *S*_BJH_ and *V*_BJH_ increases to 27.016 m^2^/g and 0.026 cm^3^/g, showing that the huge number of nanopores appear on the CIP.

**Table 2:** Comparison of particle surface characteristics.

Particle	*S*_BET_ (m^2^/g)	*S*_BJH_ (m^2^/g)	*V*_BJH_ (cm^3^/g)
CIP	0.474	0.445	0.001
HCIP	37.773	27.016	0.026

**Figure 4:**
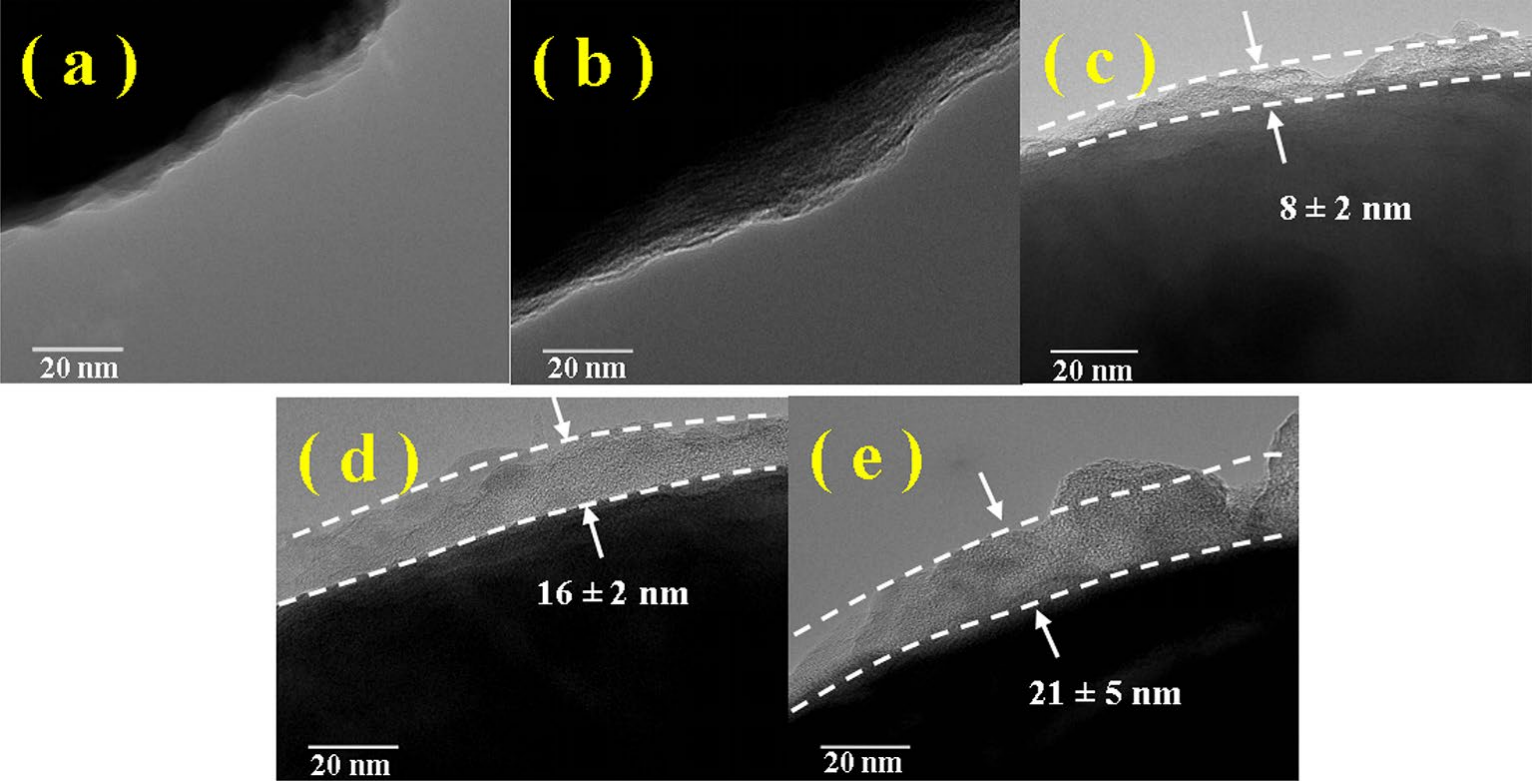
TEM images of the particles: (**a**) CIP, (**b**) HCIP, (**c**) HCIP-BTES, (**d**) HCIP-BTES-TOS and (**e**) HCIP-BTES-DTOS.

Figure 4 presents the TEM images of the particles, which exhibits an identified boundary between the particle and background scenery (Fig. 4a and b)^33,40^. In the images of the coated particle, the black area is surrounded by a transparent gray area, which is the applied silane coupling agent^41,42^. The thickness can be identified by measuring the transparent gray zone on the surface of the particles. The shell thickness of the BTES coated particle was approximately 8 nm (Fig. 4c). After the BTES and TOS being coated, the shell thickness of the particle was approximately 16 nm (Fig. 4d); After the BTES and DTOS being coated, the shell thickness of the particle was approximately 21 nm, as shown in Fig. 4e, which increased as the chain length of the silane coupling agent increased, and the particles of different shell thicknesses were obtained.

### Coating effect of different silane coupling agents on the particles

**Figure 5:**
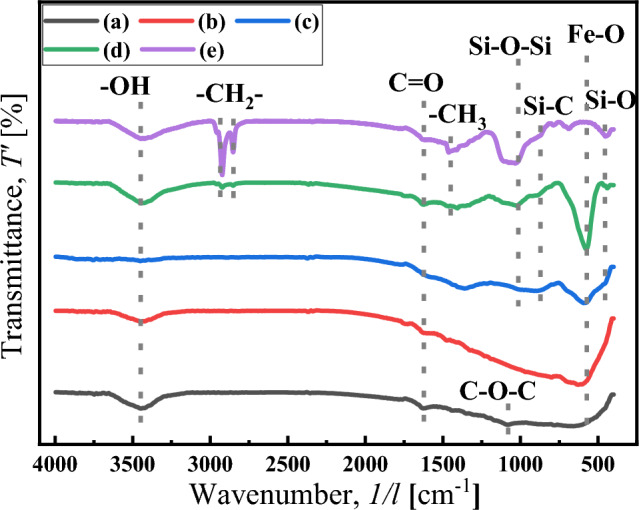
FTIR curve of particles: (**a**) CIP, (**b**) HCIP, (**c**) HCIP-BTES, (**d**) HCIP-BTES-TOS and (**e**) HCIP-BTES-DTOS.

The coating effects of the different silane coupling agents on the particles were investigated using the FTIR curves shown in Fig. 5. The peaks located at 613.32, 1089.72 and 1739.69 cm^−1^ in curve (a) and (b) indicate the existence of the Fe–O, C–O–C and C=O bonds, the peak at 3446.60 cm^−1^ indicates the existence of -OH bond, attributing to the attached water on the particle surface^25,31^. In curve (c), the peaks located at 441.67, 894.92 and 995.21 cm^−1^ confirm the existence of the Si–O, Si–C and Si–O–Si bonds in BTES and the hydrolytic functional group (Si–OH) is dehydrated and condensed on the surface of the HCIP, indicating that the coating is adsorbed on the particles by chemical bonding^25,43–45^. In curve (c), the peak located at 1465.82 cm^−1^ confirms the existence of C–H_3_ bonds, indicating that the TOS is successfully coated on the particles, and the double-layer coated particle HCIP-BTES-TOS is obtained^25^. And the vibration peak of C–H_3_ also appears in curve (e), indicating the double-layer coated particle HCIP-BTES-DTOS is obtained. Moreover, in curves (d) and (e), a strong Si–O–Si vibration absorption peak appeared at 1033.78 cm^−1^, indicating that a chemical bond was formed between the first-layer coating and the second-layer coating through silanol dehydration condensation^25,46^. The coating mechanism of the CIP particles is shown in Fig. 1b, which can be confirmed by the FTIR results.

**Figure 6:**
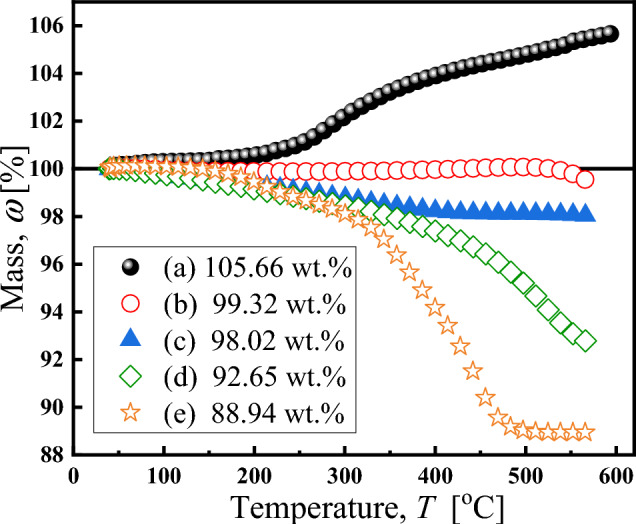
TGA curves of particles: (**a**) CIP, (**b**) HCIP, (**c**) HCIP-BTES, (**d**) HCIP-BTES-TOS and (**e**) HCIP-BTES-DTOS.

The coated mass of particle was confirmed by TGA curves (Fig. 6). Notably, The mass in curves (a) and (b) continues to increase until it reaches 500 °C, attributing to the chemical reaction between N_2_ from the testing environment and the CIP particles, and the mass of curve (a) has increased to 105.66 wt%^29^. Compared with the uncoated particles, the curve of the coated particles did not show an upward trend, indicating that the coating prevented the particles from chemically reacting with the external environment^26^. Due to the presence of the oxide layer, the mass of curve (b) increases slightly during the heating process, and the mass loss at 600 °C is 0.68 wt%, corresponding to the decomposition of particles^47^. With the increase in temperature, the curves (c), (d) and (e) decrease at about 100 °C, which was owing to the decomposition of silane molecules chemically and very little water adsorbed on the particles^48,49^. Compared with single-layer coated particles (in curves c), the mass loss of double-layer coated particles (in curves d) increased from 1.98 to 7.35 wt%, indicates the formation of a double layer coating. The quality of the double-layer coating with DTOS in sample (e) is much higher than that of the double-layer coating with TOS in sample (d), which may be due to the greater number of carbon atoms on the DTOS molecule, and the effect of methoxy group on the coating is stronger than that of ethoxy group, and thus, the sample (e) has a thicker shell than sample (d). After the particles were coated the mass loss was 1.98 wt% (in curves c), 7.35 wt% (in curves d), and 11.06 wt% (in curves e), respectively, indicating that single-layer and double-layer coated particles were obtained by the coating of utilizing silane coupling agents with different chain lengths.

### Density and magnetic properties of the particles

Table 3 lists the density and saturation magnetization of the particles. After being etched, the density of the particles decreased from 7.729 to 7.604 g/cm^3^, due to the formation of numerous porous structures on the particle surfaces, the etching slightly changed the particle density. When BTES was coated, the density of the single-layer coated particles (HCIP-BTES) decreased to 6.289 g/cm^3^, the density of double-layer coated particles (HCIP-BTES-TOS) decreased to 5.298 g/cm^3^, and the density of double-layer coated particles with longer carbon chains (HCIP-BTES-DTOS) further decreased to 5.036 g/cm^3^. This suggests that HCl etching, single-layer or double-layer coating, and silane coupling agent chain length can further affect the particle density.

**Table 3:** The coated effects of silane coupling agents with different chain lengths on the density and magnetic properties of particles.

Number	Samples	Density (g/cm^3^)	Magnetization, *M*_s_ (emu/g)
(a)	CIP	7.729	249.52
(b)	HCIP	7.604	230.59
(c)	HCIP-BTES	6.289	227.39
(d)	HCIP-BTES-TOS	5.298	189.92
(e)	HCIP-BTES-DTOS	5.036	174.74

**Figure 7:**
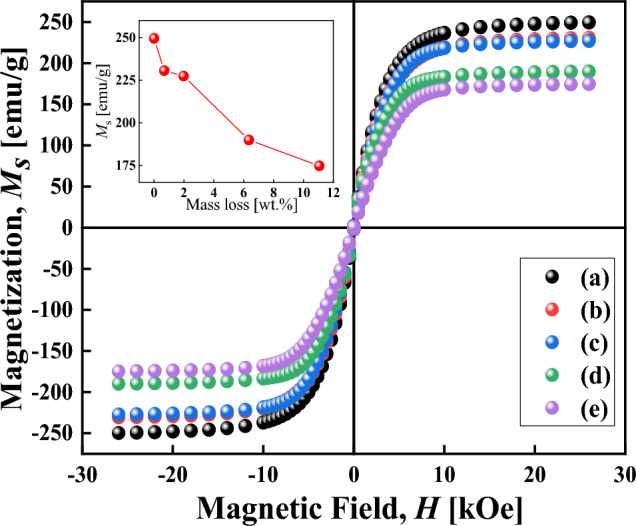
The magnetization curves of the particles: (**a**) CIP, (**b**) HCIP, (**c**) HCIP-BTES, (**d**) HCIP-BTES-TOS, and (**e**) HCIP-BTES-DTOS.

Figure 7 presents the magnetization curves of particles. By comparing curves (a) and (b), once the particles were etched, ***M***_*s*_ decreased from 249.52 to 230.59 emu/g, the saturation value was slightly different, due to the formation of oxidation layer on the surface of the HCIP^50^. The saturation magnetization of the particles decreased from 249.52 to 174.74 emu/g with the coating chain length increases, as showed in Table 4. In the insert diagram in Fig. 7, the ***M***_*s*_ of single-layer coated particles were slightly reduced, while the ***M***_*s*_ of double-layer coated particles were significantly reduced, attributing to non-magnetic molecule increase the distance between magnetic cores and reduce the interaction between particles^19,50–52^.

### The rheological properties of MRF

**Figure 8:**
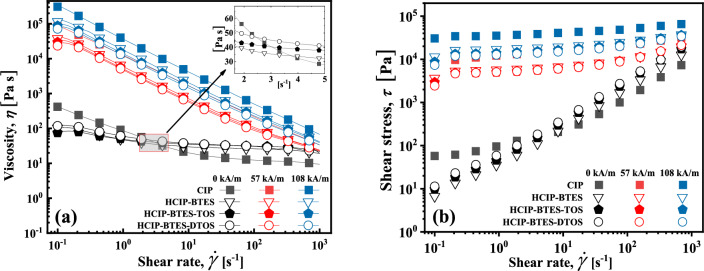
The relationship between the shear rate on viscosity and shear stress of the MRFs in different magnetic field strengths.

Figure 8a presents the correlation between shear rate and viscosity of MRF under various magnetic field strengths. Without a magnetic field, the viscosity of the MRF formed by the pure CIP significantly decreases with an increase in the shear rate, this is a typical non-Newtonian fluid, the reason for this phenomenon is the agglomeration and sedimentation of particles in MRF.

The viscosity of the MRF formed by the coated particles gently decreases with an increase in the shear rate, compared to that of the MRF composed of pure CIP, which behaves more like a Newtonian fluid. The coated silane coupling agent is conducive for improving the compatibility between the particles and the carrier medium, meanwhile the forming of a three-dimensional network structure and which prevents the sedimentation of the particles, thus the MRF prepared by the coating particles is more similar to Newtonian fluids. In addition, the viscosity of the MRF increases slightly with an increase in the coating chain length at the same shear rate, this is due to the formation of three-dimensional network structure of more complicated^53^. Under a magnetic field, the viscosity of the MRF decreases with an increase in the shear rate, demonstrating of a non-Newtonian fluids^31,54–56^.

Under the magnetic field of 57 kA/m, the shear stress of all the MRFs in Fig. 8b increases by about 100 times compared to that without a field, this is due to the formation of chain structure of magnetic particles along the direction of magnetic field. Additionally, the shear stress of MRF prepared by pure CIP is 5 times higher than that of the sample prepared by the coated particles, and the stress of MRF decreases with the increase of coating thickness. This is owing to that the magnetic saturation of the particles decreased by the coating of silane coupling agent, which is consistent with the VSM curve Equation ^24^. With the further increase of the magnetic field from 57 kA/m to 108 kA/m, although the value of the magnetic field doubled, the shear stress of MRFs only increases by about 5 times compared with the magnetic field strength of 57 kA/m, and the shear stress of MRFs prepared by coated particles are still 5 times lower than the MRF prepared by pure CIP particles. At high magnetic field intensity, the influence of magnetic field on MRF shear stress decreases gradually. And the reduction of field shear stress caused by coating can be improved by increasing the magnetic field.

**Figure 9:**
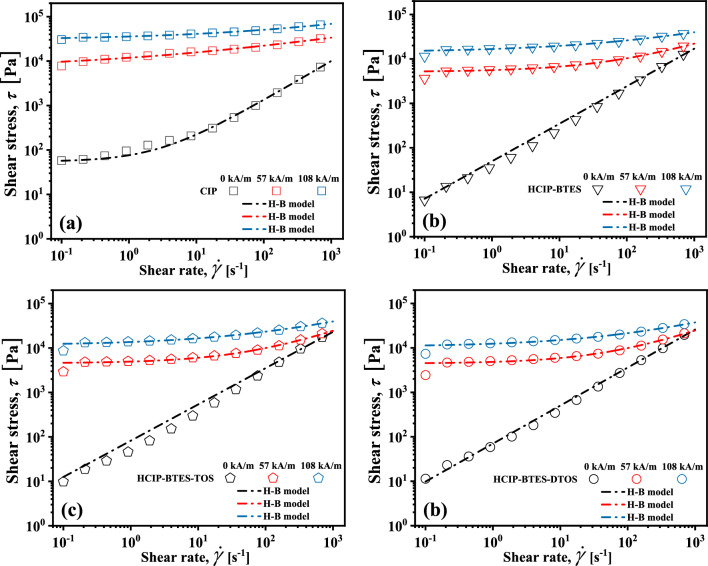
The shear stress of MRFs fitted by H–B model.

The typical Herschel–Bulkley rheological model (H–B) is adopted to calculate the experimental data of the obtained dynamic yield stress of MRF, and the dynamic stress ***τ***_y_ related to magnetic field, and the fitting curve and experimental results are plotted together in Fig. 9. The equation of H–B is as follows:

1 $$\tau = K \cdot \dot{\gamma }^{n} + \tau_{y}$$

where, ***K*** is the consistency index, and ***n*** is the non-Newtonian index.

The calculated values are shown in Table 4 and the fitted parameter values can be seen according to the data in the table. The parameter ***n*** of MRFs decreases with increase of magnetic field strength, the pseudoplastic behavior of MRFs becomes stronger. The ***n*** tends to increase with the increase of coating chain length under the magnetic field, indicate that the pseudoplastic behavior of MRFs becomes weaker. Without the magnetic field, the dynamic stress ***τ***_y_ significantly decreases after CIP is coated, which is because the coating prevents the agglomeration of particles. However, with the increase of the coating chain length, the entanglement between the coating molecules and the carrier medium is enhanced, and a stronger three-dimensional network structure is formed in the carrier medium, resulting in a trend that the ***τ***_y_ decrease and then increase. And the value of ***τ***_y_ decreases with the increase of the coating thickness, which is caused by the decrease of the saturated magnetization of particles coated. Moreover, the ***τ***_y_ of the MRF with the longest chain coating particle is reduced by about 3 times compared with the MRF prepared by CIP under the magnetic field strength of 108 kA/m^33^.

**Table 4:** The rheological parameters were obtained by fitting the dynamic yield stress with the H–B model.

Sample	Parameter	*H* = 0 (kA/m)	*H* = 57 (kA/m)	*H* = 108 (kA/m)
CIP	*n* [-]	0.89	0.24	0.23
	*K* [Pa s^n^]	22.01	5235.12	6010.77
	τ_y_ [Pa]	54.21	6640.69	29,704.18
HCIP-BTES	*n* [-]	0.84	0.51	0.33
	*K* [Pa s^n^]	49.83	500.69	2584.14
	τ_y_ [Pa]	34.04	5092.09	14,118.67
HCIP-BTES-TOS	*n* [-]	0.82	0.56	0.37
	*K* [Pa s^n^]	82.14	406.37	2112.50
	τ_y_ [Pa]	38.89	4537.80	11,524.31
HCIP-BTES-DTOS	*n* [-]	0.86	0.57	0.39
	*K* [Pa s^n^]	70.35	399.31	1775.68
	τ_y_ [Pa]	41.31	4441.87	10,660.16

### Dispersion stability of the MRF

**Figure 10:**
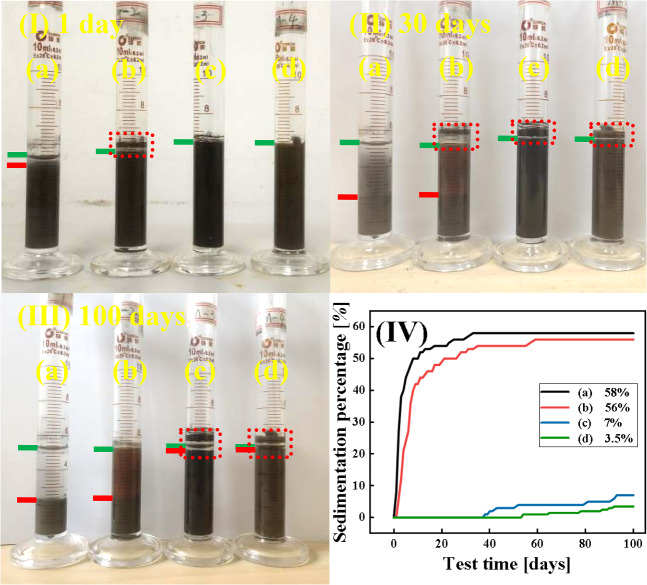
The static settlement of the samples at: (**I**) 1 d, (**II**) 30 d, (**III**) 100 d, (**IV**) curves along with time (a) CIP, (b) HCIP-BTES, (c) HCIP-BTES-TOS and (d) HCIP-BTES-DTOS.

In Fig. 10, photos (I), (II), and (III) of the MRF were captured after 1, 30, and 100 days of static settlement. In the photographs, solid residue adhered to the tube above the green line becomes visible subsequent to the decline of the MRF, attributing to the release of air that entered during mechanical agitation. The region between the green line and the red line illustrates the supernatant following the settlement of the MRF. After only 1 d of sedimentation, obvious stratification was observed in sample (a) and (b), due to the density difference between the particles and the carrier medium, and the limited reduction in particle density of the short-chain silane coupling agent coating in sample (b). After 30 d of sedimentation, a large number of particles in sample (a) and (b) have settled at the bottom of the tube, and no obvious stratifications were in sample (c) and (d). After 100 d of sedimentation, the sedimentation percentage of sample (c) was higher than that of sample (d), this is due to the better dispersion stability of long-chain than short-chain silane coupling agents. The sedimentation percentage of the MRF is expressed as follows^57^:

2 $$c = \frac{a}{a + b} \times 100\%$$

where *c* is the sedimentation percentage, *a* is the volume of the supernatant, and *b* is the volume of the underlying magnetic particles.

The variation of sedimentation rate over time is shown in Fig. 10**(IV)**. The final sedimentation percentage of sample (a) was 58% and tended to stabilize on the 30th day. The sedimentation percentage of sample (b) was 52% after 100 days of sedimentation, indicating that the single-layer coating on the particles had a small effect on the dispersion stability of the MRF.

The sedimentation percentage of sample (c) and (d) were 7% and 3.5% after 100 d of sedimentation, respectively, compared with the single coated sample (b), the sedimentation stability of the double coated sample was improved by about 94%, and the dispersion stability of sample d with longer chain coated particles was better as the coating chain length increased. Plachy et al. indicated that this is owing to the reduction in the density difference between the particles and the carrier medium^33^. In addition, Lopez-Lopez et al. reported that the coating of particles enhanced the spatial repulsion and prevented irreversible agglomeration between the particles, thus enhancing the dispersion stability of the MRF^58^.

**Figure 11:**
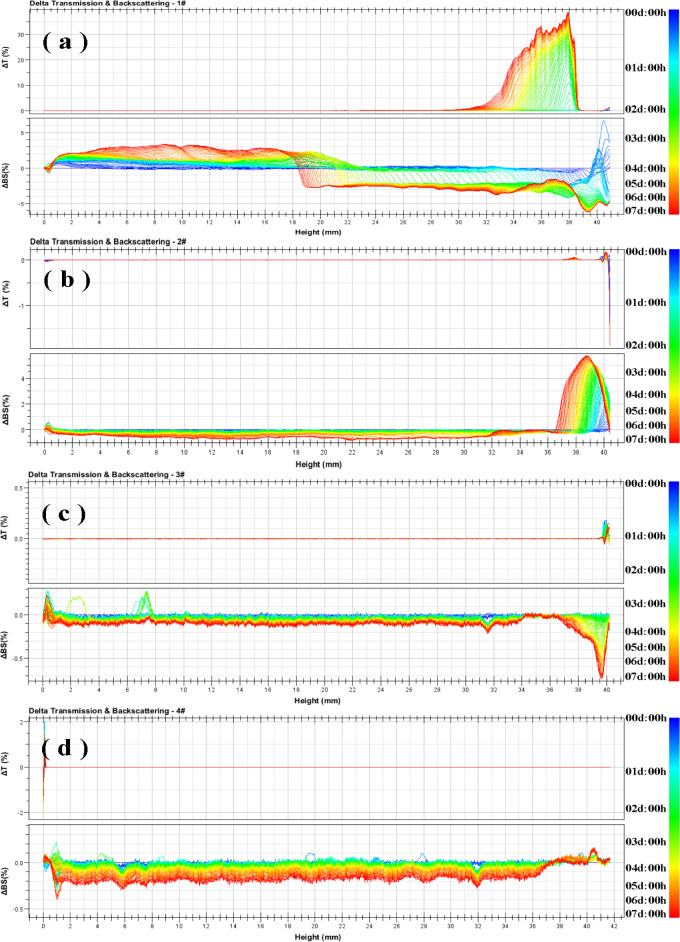
Typical Turbiscan spectra of MRFs: (**a**) CIP, (**b**) HCIP-BTES, (**c**) HCIP-BTES-TOS, and (**d**) HCIP-BTES-DTOS.

Figure 11 shows the light intensity of four MRFs as measured by Turbiscan-Lab. The measurement probe is perpendicular to the direction of the sample cell, the MRFs are scan at a height of 0–40 mm, the data of transmission (T) and backscattering (BS) are obtained every 40 μm. As shown in Fig. 11, the bottom of the sample pool was 0 mm on the left side of the x-axis, and the top of the sample pool was 40 mm on the right side. The change in line color from blue to red represents the change in test time from 0 to 7 days. In the previous reports, the ΔBS was used to study the dispersion system with high particle concentration, so as to obtain the instability information of the dispersion system and the change of the spectrum with significant reduction indicates the clarification of the sample^59^.

**Figure 12:**
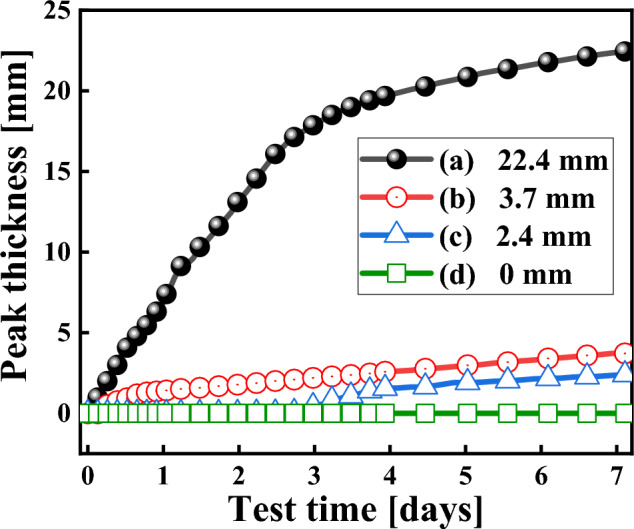
Variation in the peak thicknesses of the particles within seven days: (**a**) CIP, (**b**) HCIP-BTES, (**c**) HCIP-BTES-TOS and (**d**) HCIP-BTES-DTOS.

Figure 12 presents the migration curves of the peak thicknesses of the particles. Combine to Fig. 11, The peak thickness of particles in sample (a) was 22.4 mm, and ΔBS changed from a negative peak to a positive peak at 18 mm in Fig. 11, indicating that the sedimentation position of the sample was at 18 mm of the sample height. he peak thickness of particles in sample (b) was 3.7 mm, and ΔBS showed a steep peak drop at 36 mm, indicating the sedimentation position of the sample. The particle sedimentation value after single-layer coated particles (in sample b) is smaller than that of pure CIP (in sample a), indicating that the coating has a positive effect on the dispersion stability of MRF. Notably, ΔBS in sample (b) showed an opposite direction compared to other samples owing to the fact that the supernatant presented yellowish brown (as shown in sample b in Fig. 10), the colored supernatant increased the refractive index of the light wave. Therefore, the change of its ΔBS also indicates a clear sedimentation of particles at this position. The peak thickness of particles in sample (c) was 2.4 mm, and the negative peak of ΔBS gradually weakens at 37 mm, it indicates that the dispersion stability of sample (c) with double-layer coated particles is higher than that sample (b) with single-layer coated particles. The peak thickness of particles in sample (d) was 0 mm, and the ΔBS in the spectrogram did not change greatly, the results show that the dispersion stability of the sample (d) with long alkyl chains of double-layer coated particles is better than that of the sample (c) with short chains of double-layer coated particles.

Table 5 presents the peak thickness and sedimentation rate of MRFs after seven days. According to Table 5 the maximum sedimentation rate of the MRF prepared with pure CI particles was 0.149 mm/h, and the sedimentation rates of the MRFs gradually decreased to 0.019 mm/h, 0.017 mm/h and 0 mm/h as the coating chain length on the particles increased. The dispersion stability of the MRF can be significantly improved, which is primarily attributed to the following factors: with larger specific surface area, more –OH functional groups appear on porous CIP surfaces etched by HCl, the existence of the coating reduces the density difference between the particles and carrier medium, increases the compatibility between particles and the base load fluid, and forms a three-dimensional network structure in the carrier medium to prevent the agglomeration of particles^19,27,33,53,58^.

**Table 5:** The density and migration rate of particles after 7 d of sedimentation.

Samples	Density (g/cm^3^)	Peak thickness (mm)	Sedimentation rate (mm h^−1^)
CIP	7.729	22.4	0.149
HCIP-BTES	5.489	3.7	0.019
HCIP-BTES-TOS	5.298	2.4	0.017
HCIP-BTES-DTOS	5.036	0	0

## Conclusions

In this study, the CIP was etched with HCl to generate porous structure and modified the surface properties of the particles. Different chain length of silane coupling agent were chemically coated on the CIP to form single-layer or double-layer coated particles. As the chain length of the silane coupling agent increases, the non-magnetic coating mass of particles is improved, the particle density and saturation magnetization gradually decreased, and particles shells with different thicknesses were obtained; without a magnetic field, the viscosity of MRF increases slightly due to the formation of a complex three-dimensional network structure; under a magnetic field, the viscosity of the MRF decreased, the coated non-magnetospheric increased the distance between the magnetic cores and reduced the magnetic interaction force between particles; the sedimentation percentage of MRF after 100 days of the settlment decreased from 58 to 3.5%, the migration distances of the MRFs within 7 days were 22.4, 3.7, 2.4, and 0 mm, with particle sedimentation rates of 0.149, 0.019, 0.017, and 0 mm/h, respectively. The etching of CTP by HCl and short-chain coupling agent with multi-functional groups used as the first coating are effective manners for improving the coating effect of CIP, and the MRF with high stability are obtained and which will drive technological innovation in related fields such as advanced manufacturing, medical equipment and robotics.

## Data Availability

The datasets generated and/or analysed during the current study are not publicly available due to that the commercial restriction but are available from the corresponding author on reasonable request.
